# Nicotine Modulates MyD88-Dependent Signaling Pathway in Macrophages during Mycobacterial Infection

**DOI:** 10.3390/microorganisms8111804

**Published:** 2020-11-17

**Authors:** Dania AlQasrawi, Saleh A. Naser

**Affiliations:** Division of Molecular Microbiology, Burnett School of Biomedical Sciences, College of Medicine, University of Central Florida, Orlando, FL 32816, USA; daniaqasrawi@Knights.ucf.edu

**Keywords:** Nicotine, MAP, Macrophages, TLR2, MyD88, Crohn’s disease

## Abstract

Recently, we reported that cigarette smoking, and especially nicotine, increases susceptibility to mycobacterial infection and exacerbates inflammation in patients with Crohn’s disease (CD). The macrophagic response to *Mycobacterium avium* subspecies *paratuberculosis* (MAP) in CD and *Mycobacteria tuberculosis* (MTB) continues to be under investigation. The role of toll-like-receptors (TLRs) and cytoplasmic adaptor protein (MyD88) in proinflammatory response during Mycobacterial infection has been suggested. However, the mechanism of how nicotine modulates macrophage response during infection in CD and exacerbates inflammatory response remain unclear. In this study, we elucidated the mechanistic role of nicotine in modulating MyD88-dependent/TLR pathway signaling in a macrophage system during mycobacterial infection. The data demonstrated that MAP infection in THP-1 derived macrophages was mediated through TLR2 and MyD88 leading to increase in *IL-8* in expression and production. On the other hand, LPS-representing, Gram-negative bacteria mediated macrophage response through TLR4. Blocking TLR2 and TLR4 with antagonists voided the effect of MAP, and LPS, respectively in macrophages and reversed response with decrease in expression of *iNOS*, *TNF-α* and *IL-8*. Interestingly, nicotine in infected macrophages significantly (1) downregulated TLR2 and TLR4 expression, (2) activated MyD88, (3) increased M1/M2 ratio, and (4) increased expression and secretion of proinflammatory cytokines especially *IL-8*, as seen in CD smokers. We also discovered that blocking macrophages during MAP infection with MyD88 antagonist significantly decreased response which illustrates the key role for MyD88 during infection. Surprisingly, dual treatment of MAP-infected macrophages with MyD88 antagonist and nicotine absolutely impaired immune response and decreased MAP viability, which clearly validate the inflammatory role of nicotine in macrophages through TLR2/MyD88 pathway during infection. This is the first report to describe the mechanism by which nicotine modulates TLR2/MyDD88 and exacerbates inflammation in CD smokers associated with infection.

## 1. Introduction

Crohn’s Disease (CD) is one of the relapsing chronic diseases that affects the gastrointestinal tract. Although the etiology of CD is still vigorously debated, mounting evidence at the basic and clinical research levels has highlighted and strongly associated CD with microbial infection, immune-dysregulation, and mucosal dysfunction. Among the most debated pathogens that have been studied for their possible role in CD pathogenesis are *Mycobacterium avium* subspecies *paratuberculosis* (MAP), adherent-invasive *Escherichia coli* (AIEC), and *Klebsiella pneumoniae* [1,2,3,4]. The immune response in patients with CD is unique with clear involvement of both Th1/Th2 signaling leading to humoral and innate immune response. Innate immunity is considered an early warning system against any pathogenic attachment where macrophages play a crucial role in recognizing bacteria through their own cellular receptors such as pattern-recognition receptors (PRRs). PRRs are highly expressed in macrophages and are involved in recognition of both pathogen-associated molecular patterns (PAMPs) and damage-associated molecular patterns (DAMPs) [5]. Furthermore, toll-like receptors (TLRs) are members of the PRR family that have been identified as a central hub of pathogen recognition which results in the immune response [6]. TLRs are found in most mammals and each of them has specific transmembrane proteins with their own recognition patterns [7].While most TLRs are located on the cell surface, which plays an important role in recognition of microbial derivatives, TLR3, TLR7, and TLR9 are localized in the endosomes and bind mainly to microbial nucleic acids [8,9]. TLRs recognize their ligands as heterodimers in TLR1/TLR2 and TLR6/TLR2, homodimers in TLR3, TLR4, TLR5 and TLR9, and monomers in TLR7 and TLR8 (Table 1). Table 1 summarizes key TLRs and their location and functions.

Recently, TLRs have been causally linked to play a role in the pathogenesis of inflammatory bowel disease (IBD). Specifically, macrophages in CD patients seem to have high expression of TLRs compared to those in patients with ulcerative colitis (UC). We believe the difference in macrophage TLRs expression level among IBD subsets is most likely due to the association of CD with intracellular infection. MAP is the causative agent of CD-like disease in animals and has been widely reported to be involved in more than 50% of CD cases [2,10]. It is still unclear which TLRs are involved in mycobacterial infection. However, mycobacterial cell wall components such as lipoarabinomannan (LAM), similar to LPS in Gram-negative bacteria, have been suggested to interact with TLRs during infection [11]. TLR9 has been reported to recognize and interact with mycobacterial DNA in phagosomes following phagocytosis [11,12,13]. On the other hand, TLR4 has been confirmed to play a role in recognition of Gram-negative bacteria through interaction with LPS [12].

TLR activation affects downstream signaling pathways including major adaptor proteins such as myeloid differentiation primary response 88 (MyD88) [14]. All TLRs, except TLR3, require MyD88 to mediate signal transduction pathways in response to inflammatory triggers in macrophages [14]. The MyD88-dependent signaling pathway was reported to be similar to the IL-1 receptor pathways in activation of IRAK-1, leading to activation of the MAP-kinase and NF-κB pathways [14,15,16]. We speculated that there is a link between the high expression of TLRs in macrophages with the MyD88-dependent pathway and the proinflammatory response during infection in Crohn’s disease. If confirmed, the outcome should support a microbial etiology in CD pathogenesis.

Autophagy is an effector mechanism used by immune cells to eliminate intracellular bacteria [17]. It has been reported to occur during Mycobacterium tuberculosis infection. Recent studies reported an interplay between vitamin-D-dependent antimicrobial response, TLR2, and the autophagy pathway leading to enhanced macrophage response against M. tuberculosis infection [17,18,19,20]. Specifically, they reported that 1,25 dihydroxyvitamin D_3_ (1,25D_3_) induces the production of cathelicidin and activation of the autophagy pathway in MTB-infected macrophages resulting in protective immune response against M. tuberculosis infection [11]. Given the 90% homology between MTB and MAP, we proposed that the TLR2-signaling pathway plays a critical role in the macrophage response during MAP infection in CD.

As we reported recently, cigarette smoke (CS) and nicotine exacerbate inflammation in CD smokers and provide a protective anti-inflammatory response in UC smokers [21]. Specifically, we demonstrated that successive inflammatory response in CD smokers is due to the presence of MAP infection in these patients [21,22]. We also elucidated the role of α7nAChR in mediating the nicotine effect in MAP-infected macrophages [21]. We reported that nicotine exacerbates MAP infection in macrophages by upregulation of *iNOS*, increasing M1/M2 ratio and proinflammatory cytokines such as tumor necrosis factor alpha (*TNF-α*) and IL-6 [21]. Despite the new findings by this group, there is a big gap in the literature about how nicotine modulates macrophage recognition patterns including TLRs and MyD88 signaling during mycobacterial infection. Specifically, it is not understood how nicotine affects MAP recognition and infection in macrophages causing further inflammation and worsening symptoms in CD smokers. This study is focused on the elucidation of the mechanistic role of nicotine in modulating TLR and the MyD88-dependent signaling pathway in MAP-infected macrophages. We used in this study THP-1 differentiated macrophages, which are easy to obtain, differentiate and polarize. M1 and M2 THP-1 macrophages have the same expression profiles as polarized primary intestinal macrophages. We also investigated the interplay between nicotine, TLR2 and TLR4 for their reported role in infection by gram-negative and -positive bacterial infection, and subsequent effect on MyD88 signaling, inflammatory response, and bacterial load in infected macrophages.

## 2. Materials and Methods

### 2.1. Culture Conditions of THP-1 Macrophages

THP-1 monocytes (ATCC TIB-202) were cultured as described earlier [23]. Briefly, cells were maintained in RPMI-1640 (ATCC 30-2001) growth medium with 10% fetal bovine serum (FBS; Sigma Life Science, St. Louis, MO, USA). The cells then were maintained in a humidified 5% CO_2_ incubator at 37 °C until they reached 80% confluency. Then, cells were activated by adding 50 ng/mL of phorbol 12-myristate 13-acetate (PMA; Sigma Life Science, St. Louis, MO, USA) for 24 h. All experiments were performed in duplicates and done within eight passages.

### 2.2. Infection and Treatment of THP-1 Macrophages

Infection: Activated THP-1 cells (M0 macrophages) were plated at a density of 3 × 10^5^ cells in 12-well plates. Cells were infected for 24 h at 37 °C in 5% CO_2_ with 1 × 10^7^ CFU/mL of MAP strain UCF4 (a clinical strain isolated from CD patient), *M. tuberculosis* ATCC HR237, or *K. pneumoniae* ATCC13883. Bacterial lipopolysaccharide (LPS) from *E. coli* strain O111.B4 (Sigma Life Science, St. Louis, MO, USA, 5 µg/mL), and uninfected cells were used as a control.

Treatment with nicotine: activated THP-1 macrophages were treated with nicotine (Sigma Life Science, St. Louis, MO, USA, 4 µg/mL). Cells were incubated for an additional 24 h at the same conditions.

Receptor inhibitors: To determine the role of TLR and MyD88 in the cellular recognition of MAP and its interplays with nicotine, activated THP-1 macrophages were pretreated with MMG11 (TLR2 antagonist; Bio-Techne, Minneapolis, MN, USA), TLR4-IN-C34 (TLR4 inhibitor; Sigma-Aldrich, St. Louis, MO, USA) and T6167923 (MyD88 inhibitor; Aobious, Waltham, MA, USA) (Table 2) separately at room temperature for 30 min before MAP infection. All experiments were performed in duplicate.

### 2.3. Measurement of Relative Gene Expression Using RT-PCR

RNA isolation: After 24 h treatment, cell pellets were collected in 2 mL-microcentrifuge and then suspended in 500 µL of TRIzol^®^ reagent (Invitrogen, Carlsbad, CA, USA). A 125 µL of chloroform was added to each tube, mixed and incubated at room temperature 5 min. Following centrifugation at 10,000 rpm for 5 min, the aqueous phase was transferred to a new tube and mixed with 275 µL isopropanol. Following centrifugation at 14,000 rpm and 4 °C for 15 min, RNA pellets were isolated, washed in 500 µL of 75% ethanol, air-dried, and then dissolved in 15 µL of Tris-EDTA (TE) buffer. RNA was stored at −80 °C until further use.

cDNA Synthesis: Reverse transcription reaction was carried out by adding 800 ng of RNA in 0.25-mL microtubes containing 20 µL of PCR reaction; 4 µL iScript™ reverse transcription (Bio-Rad^®^, Hercules, CA, USA) and up to 0.20 mL RNAse-free water. The reaction was performed using MyGene Series Peltier Thermal Cycler under the following conditions: 5 min at 25 °C, 20 min at 46 °C and 1 min at 95 °C.

RT-PCR: Gene expression analysis was performed as described earlier [22]. Briefly, a total volume of 20 µL reaction mixture containing 1 µL of cDNA (30 ng/µL), 10 µL of Fast SYBR Green Mastermix (Thermo Fisher Scientific, Waltham, MA, USA), 1 µL of forward primer, 1 µL of reverse primer of either TLR2, *TLR4*, MyD88, *IL-8*, *iNOS*, *MMR*, *TNF-α* or *IL-10* (Thermo Fisher Scientific, Waltham, MA, USA) (Table 3), and 7 µL of RNAse-free water, were prepared in a 96-well Microamp RT-PCR reaction plate and placed analyzed using 7500 Fast Real- Time PCR System (Applied Biosystems, Foster City, CA, USA). Housekeeping *GAPDH* primer was used to obtain baseline CT readings. Relative mRNA expression as fold change was calculated by using equation 2^(−∆∆CT)^, where ∆CT = [(sample RT-PCR CT value) − (*GAPDH* CT baseline value)] whereas ∆∆CT = [∆CT_Treated_ − ∆CT_Untreated_].

### 2.4. Measurement of Cell Receptors

For cell receptors, cell pellets were incubated with iced RIPA buffer (Thermo-Fisher, Waltham, MA, USA) for 15 min, then centrifuged at 14,000 rpm for 20 min at 4 °C. Supernatant containing cell lysates were analyzed using ELISA kits: TLR2 (RayBiotech, Norcross, GA, USA) and *TLR4* (RayBiotech, Norcross, GA, USA) following manufacturer’s instructions. All ELISA experiments were done in duplicates.

### 2.5. MAP Viability Assay

To determine if TLR2 and MyD88 have a role in MAP viability in macrophages, THP-1 macrophages were blocked by either MMG (5 µg/mL) or T6167923 (5 µg/mL) followed by MAP infection. After 24 h of infection, THP-1 macrophages were washed twice with PBS to remove extracellular bacteria, then THP-1 macrophages were collected at three time points: 0, 24, and 84 h. The cells were lysed using iced RIPA buffer (Thermo-Fisher, Waltham, MA, USA), and then the samples were centrifuged at 14,000 rpm for 20 min at 4 °C. Supernatants were collected and the LIVE/DEAD™ BacLight™ Bacterial Viability Kit (ThermoFisher Scientific, Waltham, MA, USA) was used according to the manufacturer’s protocol as described earlier [24]. Briefly, 100 µL of each sample was loaded into separate wells of a 96-well flat-bottom microplate in triplicate and mixed with 100 µL of fluorescent stain solution. The plate was then incubated for 15 min at room temperature (RT) in the dark. Fluorescence was measured using a microplate reader at 530 nm to determine the proportion of live bacteria (green), and at 630 nm to determine the proportion of dead bacteria (red). Data was analyzed by generating a standard plot using fluorescence measurements of five different proportions of live to dead MAP including 0:100, 10:90, 50:50, 90:10, and 100:0. A graph was generated, and the equation of the least-squares fit of the relationship between percent live bacteria (x) and green/red fluorescence ratio (y) was used to calculate bacterial viability of the MAP-infected macrophages. The experiments were done in triplicate.

### 2.6. Statistical Analysis

Statistical analysis was performed by using GraphPad Prism 7.02 software. Significance among experiments was assessed by an unpaired two-tailed t test at *p* < 0.05 and a 95% confidence interval (CI). All data collected in this study were pretested for normal distribution using the Kolmogorov–Smirnov normality test. Data is shown as (mean ± SD). *p*-values < 0.001 are also mentioned when achieved.

## 3. Results

### 3.1. TLR2/TLR4 Expression during Infection in Macrophages

**Figure 1:**
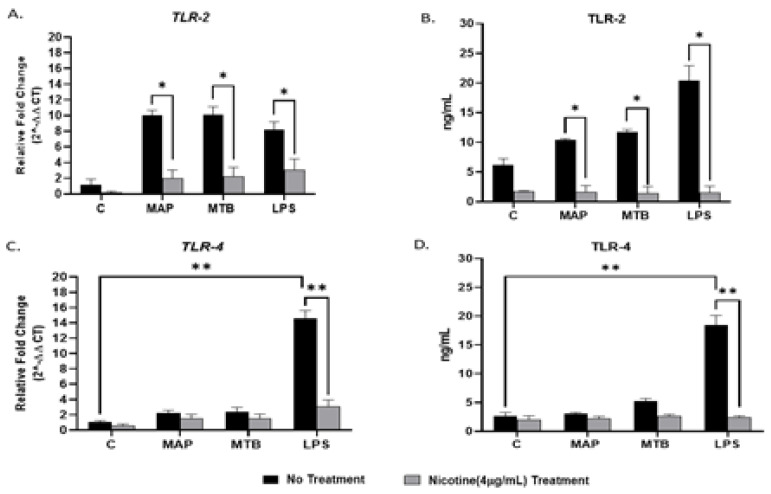
Effect of nicotine on the TLR2/*TLR4* expression on infected macrophages. THP-1 PMA differentiated macrophages were infected with MAP and MTB, or treated with LPS for 24 h, then treated with nicotine (4 µg/mL). Expression of TLR2 (**A,B**) and *TLR4* (**C,D**)were measured by RT-PCR and ELISA, respectively. All experiments were performed in duplicates. MAP: *Mycobacterium avium paratuberculosis.* MTB: *Mycobacteria tubercalusis.* LPS: lipopolysaccharide derived from *Escherichia coli* ATCC 8739. * *p* < 0.05, ** *p* < 0.001.

In order to determine the effect of bacterial infection on TLR2/*TLR4* expressions on macrophages, we infected PMA-activated THP-1 with MAP for 24 h. MTB and LPS were also used as controls. Infection of macrophages with Mycobacteria increased TLR2 in gene and protein levels, respectively (MAP: 10.03 ± 0.64, MTB: 10.11 ± 1.02 vs. uninfected: 1.20 ± 0.67; *p* < 0.05 for gene; Figure 1A), (MAP: 10.42 ± 0.20, MTB: 11.71 ± 0.42 vs. uninfected: 6.15 ± 1.06; *p* < 0.05 for protein; Figure 1B). It is worth mentioning that the effect of mycobacterial infection on *TLR4* expression was not significant when compared with uninfected untreated cells (Figure 1C). On the other hand, LPS elevated the expression of *TLR2/TLR4* on macrophages at the gene level. However, the significant effect was observed more in *TLR4* when compared with the negative control (LPS: 14.54 ± 1.07 vs. uninfected cells: 1.02 ± 0.23; *p* < 0.001) (Figure 1A,C). A similar trend was also observed at the protein level (Figure 1B,D).

### 3.2. Nicotine Modulates TLR2/TLR4 Expression in Macrophages during Infection

To determine if nicotine modulates the expression of TLR2/*TLR4* in infected macrophages, we treated infected macrophages with 4 µg/mL of pure nicotine for 24 h followed by RT-PCR and ELISA evaluation of the expression and protein levels TLR2/*TLR4*. As shown in Figure 1A, adding nicotine treatment to infected macrophages significantly decreased TLR2 expression across all infections (MAP + nicotine: 2.0 ± 1.04 vs. MAP alone: 10.03 ± 0.64; *p* < 0.05), (MTB+ nicotine: 2.24 ± 1.14 vs. MTB alone: 10.11 ± 1.02; *p* < 0.05), or (LPS + nicotine: 3.07 ± 1.40 vs. LPS alone: 8.19 ± 1.01; *p* < 0.05). The findings were consistent with results from protein level analysis (Figure 1B) (MAP + nicotine: 1.59 ± 1.13 vs. MAP alone: 10.42 ± 0.20; *p* < 0.05), (MTB+ nicotine: 1.44 ± 1.10 vs. MTB alone: 11.71 ± 0.42; *p* < 0.05), or (LPS + nicotine: 1.52 ± 1.10 vs. LPS alone: 20.45 ± 2.4; *p* < 0.05). Similarly, *TLR4* expression and protein levels corroborated those of TLR2 with lowest values reported after nicotine treatment of infected cells (Figure 1C,D).

### 3.3. Nicotine/TLR2 Interaction Modulates MyD88 Signaling in Macrophages during Infection

**Figure 2:**
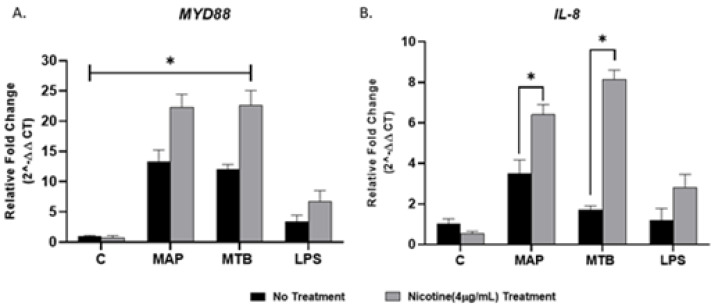
Effect of nicotine on MyD88 signaling and subsequent *IL-8* expression in macrophages during infection. THP-1-PMA-differentiated macrophages were infected with MAP and MTB, or treated with LPS for 24 h, then treated with nicotine (4 µg/mL). Expression of MyD88 (**A**) and *IL-8* (**B**) were measured by RT-PCR. All experiments were performed in duplicates. MAP: *Mycobacterium avium paratuberculosis.* MTB: *Mycobacteria tubercalusis.* LPS: lipopolysaccharide derived from *Escherichia coli* ATCC 8739. * *p* < 0.05.

To determine if MyD88 is the main adaptor protein in TLR2 signaling and to examine the effect of nicotine on MyD88 signaling and subsequent proinflammatory cytokine *IL-8* in macrophage during infection, we measured expression of MyD88 and *IL-8* following nicotine treatment of MAP-infected macrophages. MTB and LPS were also used as controls. Compared with MAP infection alone, nicotine upregulated MyD88 and *IL-8* expression, the latter showed more noticeable change of 2.5-fold (Figure 2A,B) (MAP + nicotine: 22.3 ± 2.09 vs. MAP alone: 13.39 ± 1.80; *p* < 0.05, for MyD88), (MAP + nicotine: 6.40 ± 0.52 vs. MAP alone: 3.52 ± 0.62; *p* < 0.05, for *IL-8*). However, a significant elevation in MyD88 expression was observed in all mycobacterial infection without nicotine treatment compared to untreated uninfected cells, *p* < 0.05. Interestingly, there was no clear change in MyD88 and *IL-8* expression when macrophages were treated with LPS (Figure 2A,B).

### 3.4. Antagonism of TLR4 Abrogates Macrophages Response during Gram Negative Bacterial Infection

**Figure 3:**
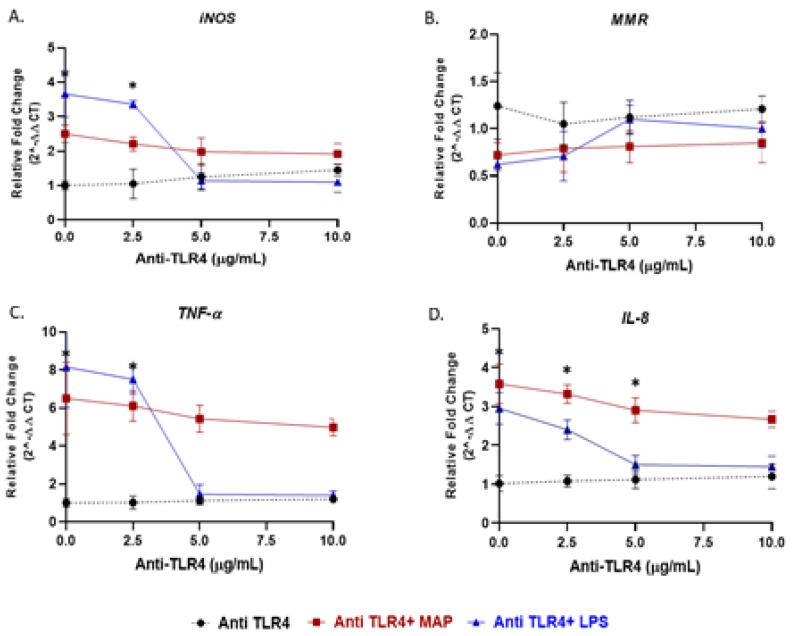
*TLR4* antagonist abrogates the macrophage response against LPS but not MAP: M1, M2, *TNF-α*, and *IL-8* studies. THP-1 PMA differentiated macrophages were infected with MAP or treated with LPS for 24 h after pretreatment with 0, 2.5, 5 and 10 µg/mL of *TLR4*-IN-C34 (Anti-*TLR4*). Expression of *iNOS* (**A**), *MMR* (**B**), *TNF-α* (**C**) and *IL-8* (**D**) were measured by RT-PCR. All experiments were performed in duplicate. * *p* < 0.05.

To validate the role of *TLR4* in macrophages response to bacterial infection, we blocked *TLR4* with *TLR4*-IN-C34, a *TLR4* antagonist. Specifically, PMA-activated THP-1 macrophages were pretreated with 0, 2.5, 5 and 10 µg/mL of *TLR4*-IN-C34, followed by either treated with LPS or infected with MAP. In absence of *TLR4*-IN-C34, LPS significantly increased *iNOS* level (3.66 ± 0.66 compared to 1.005 ± 0.125 in macrophages not treated with LPS; *p* < 0.05). Pretreated macrophages with (2.5 to 10 µg/mL) gradually neutralized the effect of LPS on i*NOS* level and reached its optimum effect at 5 µg/mL (Figure 3A). A similar trend was also observed on *TNF-α* and *IL-8* expressions (Figure 3C,D). Likewise, preconditioning cells with *TLR4*-IN-C34 (5 µg/mL) has increased M2-macrophages expression (*MMR*) (*TLR4*-IN-C34 (5 µg/mL) + LPS: 1.10 ± 0.14 vs. LPS alone: 0.62 ± 0.06 vs. *TLR4*-IN-C34 (5 µg/mL) alone: 1.12 ± 0.18; *p* < 0.05) (Figure 3B). Surprisingly, there was a modest decrease in *iNOS*, *TNF-α* and *IL-8* levels even with high concentration of *TLR4*-IN-C34 (10 µg/mL) with MAP infection (*TLR4*-IN-C34 (10 µg/mL) + MAP: 1.92 ± 0.30 vs. MAP alone: 2.5 ± 0.25; *p* < 0.05, for *iNOS*) (*TLR4*-IN-C34 (10 µg/mL) + MAP: 4.98 ± 0.45 vs. MAP alone: 6.50 ± 1.90; *p* < 0.05, for *TNF-α*) (*TLR4*-IN-C34 (10 µg/mL) + MAP: 2.67 ± 0.21 vs. MAP alone: 3.58 ± 0.51; *p* < 0.05, for *IL-8*) (Figure 3A,C,D).

### 3.5. Antagonism of MyD88 or TLR2 Alters the Level of Macrophages Response during Infection

**Figure 4:**
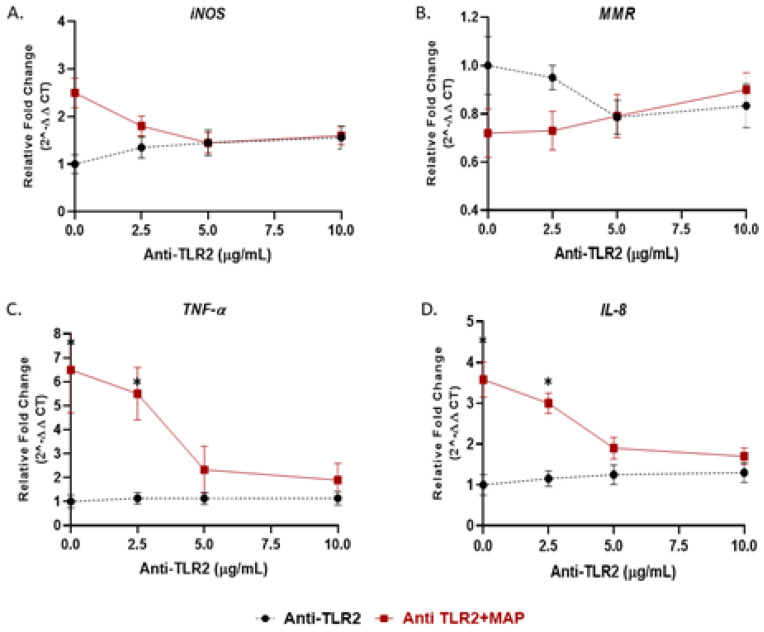
TLR2 antagonist decreases the macrophage response against MAP infection: M1, M2, *TNF-α*, and *IL-8* studies. THP-1-PMA-differentiated macrophages were infected with MAP for 24 h after pretreatment with 0, 2.5, 5 and 10 µg/mL of MMG11 (Anti-TLR2). Expression of *iNOS* (**A**), *MMR* (**B**), *TNF-α* (**C**) and *IL-8* (**D**) were measured by RT-PCR. All experiments were performed in duplicates. * *p* < 0.05.

We showed in the previous experiment that *TLR4* has no role during MAP infection; thus, we examined the effect of TLR2 and its adaptor protein (MyD88) on macrophages response during MAP infection. Activated macrophages were preincubated with 0, 2.5, 5, 10 or 20 µg/mL of either MMG11 (TLR2 antagonist) or T6167923 (MyD88 inhibitor), followed by infection with MAP for 24 h. Interestingly, MMG11 at 5 µg/mL attenuated the proinflammatory status in MAP-infected macrophages and greatest decrease was observed in *TNF-α*, falling almost fourfold compared to MAP-infected macrophages alone (MMG11 (5 µg/mL) + MAP: 2.33 ± 0.98 vs. MAP alone: 6.50 ± 1.90; *p* < 0.05) (Figure 4C).

**Figure 5:**
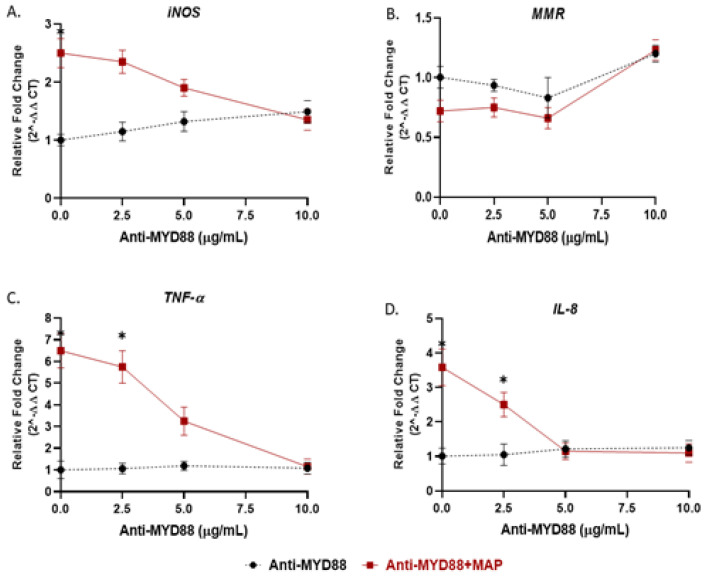
MyD88 antagonist cancels the macrophage response against MAP infection: M1, M2, *TNF-α*, and *IL-8* studies. THP-1 PMA differentiated macrophages were infected with MAP for 24 h after pretreatment with 0, 2.5, 5 and 10 µg/mL of T6167923 (Anti-MyD88). Expression of *iNOS* (**A**), *MMR* (**B**), *TNF-α* (**C**) and *IL-8* (**D**) were measured by RT-PCR. All experiments were performed in duplicate. * *p* < 0.05.

On the other hand, 10 µg/mL of T6167923 is the optimum to cancel the macrophages’ immunity against MAP infection by decreasing the expression of *TNF-α* and *IL-8* to levels similar to uninfected macrophages (T6167923 (10 µg/mL) + MAP: 1.15 ± 0.35 vs. T6167923 (10 µg/mL): 1.080 ± 0.27; *p* < 0.05, for *TNF-α*) (T6167923 (10 µg/mL) + MAP: 1.10 ± 0.27 vs. T6167923 (10 µg/mL): 1.25 ± 022; *p* < 0.05, for *IL-8*) (Figure 5C,D) and skewing macrophages toward alternative M2 phenotype (T6167923 (10 µg/mL) + MAP: 0.92 ± 0.35 vs. MAP alone: 0.71 ± 0.10 vs. T6167923 (10 µg/mL) alone: 0.90 ± 0.20; *p* < 0.05, for *MMR*) (Figure 5B).

### 3.6. Effect of Nicotine on MyD88 Signaling in Macrophages during Infection

**Figure 6:**
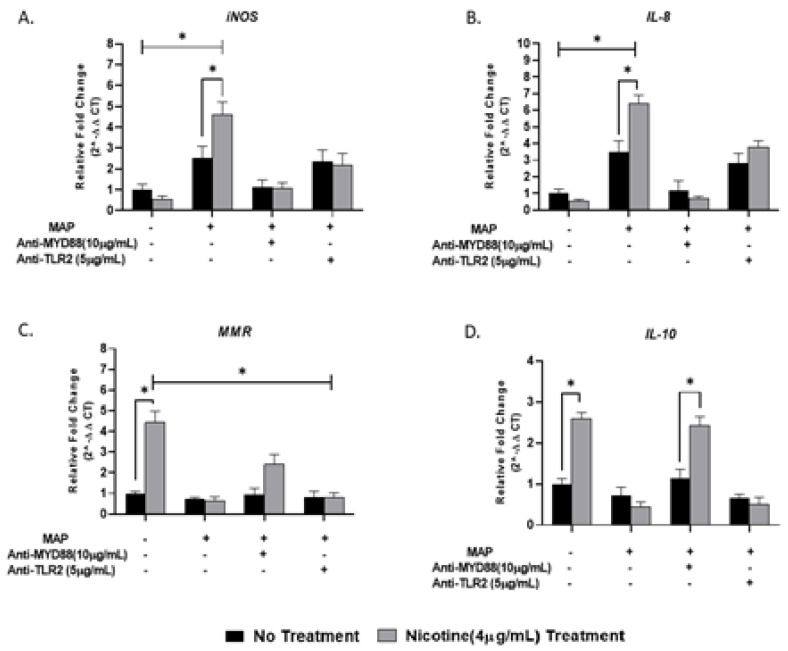
Effect of nicotine/MAP infection on inflammatory response following TLR2 and MyD88 inhibition in vitro. MAP-infected macrophages were preincubated with 10 µg/mL of T6167923 or 5 µg/mL of MMG11, following by nicotine treatment for 24 h. Expression of *iNOS* (**A**), *IL-8* (**B**), *MMR* (**C**) and *IL-10* (**D**) were measured by RT-PCR. All experiments were performed in duplicates. (**+**): Presence, (**-**): Absence. * *p* < 0.05.

To address the effect of nicotine on TLR2/MyD88 signaling in macrophages, we targeted macrophages with either 5 µg/mL of MMG11 (TLR2 inhibitor) or 10 µg/mL of T6167923 (MyD88 inhibitor) in the presence and absence of MAP infection and then subjected the cells to nicotine treatment. While the synergy of MAP and nicotine exhibited the highest levels of *iNOS* (4.62 ± 0.59) and *IL-8* (6.40 ± 0.51) compared to all other groups, blocking MyD88 or TLR2 subverted macrophages’ response to MAP by reduction of *iNOS* and *IL-8* expression to a level similar to uninfected macrophages regardless of nicotine treatment (Figure 6A,B). The opposite effect was observed in anti-inflammatory profile, using TLR2/MyD88 antagonists before MAP infection leads to shift polarization toward M2 and increase *IL-10* production (Figure 6C,D). It is worth to mention that using the MyD88 antagonist before MAP infection significantly boosted the immunosuppressive effect of nicotine on macrophages by increasing *IL-10* production 2.5-fold compared with MAP infection alone.

### 3.7. The Role of Nicotine and TLR-2/MyD88 Signaling in MAP Survival in Macrophages

**Figure 7:**
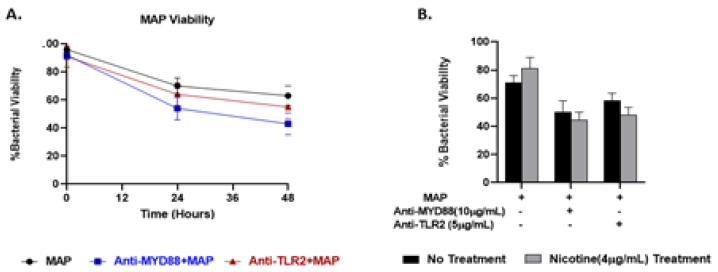
Effect of nicotine on MAP survival in macrophages blocked with TLR2/MyD88 antagonists. MAP viability was measured in infected macrophages preincubated with 10 µg/mL of T6167923 or 5 µg/mL of MMG11 after 48 hours without nicotine (**A**) and with nicotine treatment (**B**). All experiments were performed in duplicates. (**+**): Presence, (**-**): Absence.

We previously reported that exposure of MAP-infected macrophages to nicotine maintains MAP viability and increases its burden in macrophages [21]. Here we studied a possible role for TLR2 or MyD88 in MAP survival in macrophages in presence and absence of nicotine treatment. MAP viability in macrophages treated with anti-TLR2 decreased to 63% but after 48 h infection. Likewise, MAP viability in macrophages treated with anti-MyD88 decreased to 55% but after 48 h infection (Figure 7A). Nicotine treatment did not alter the viability of MAP after TLR2/MyD88 antagonists compared to no nicotine treatment (Figure 7B).

## 4. Discussion

MAP, a slow-growing intracellular bacterium capable of surviving within host macrophages, remains one of the most debated etiological agents of CD [25,26,27,28,29,30]. Macrophages play an essential role during the early immune response against MAP infection. Several pattern recognition receptors such as TLRs have been involved in mycobacterial recognition in macrophages [17]. Among all cell surface TLRs, TLR2 and *TLR4* have been identified to interact with pathogens through specific ligand binding [17]. On the other hand, nicotine, a lipophilic compound, is considered the most active ingredient in tobacco leaves [31]. We have reported recently that nicotine immunosuppressive properties in macrophages were lost when macrophages were infected with MAP, *M. tuberculosis* or treated with LPS. In fact, in our study we showed that nicotine seems to exacerbate inflammatory response during infection [21]. These intriguing findings should explain the proinflammatory role of cigarette smoking in patients with CD which is contrary to smokers with UC. This rationale is based on the extensive literature that associates CD with microbial infection. Not much is found in the literature on the mechanism involved in how nicotine exacerbation of inflammatory response in infected macrophages. This led us to study a possible interaction between nicotine and TLR2/*TLR4* in macrophages during infection.

It was estimated that each tobacco cigarette contains an average of 8.4 mg of nicotine and the average nicotine concentration in a smoker’s venous blood is approximately 2 µg/mL [32]. Of course, these estimates are subjective since smokers vary in how many cigarettes they use daily. Interestingly, 80–90% of nicotine is absorbed by most tissues that have high affinity for nicotine, which results in several times greater nicotine concentration than in venous blood. Moreover, the first-pass metabolism of nicotine in the liver contributes to tenfold increase in nicotine concentration in atrial blood compared to venous blood [32]. In this study, we tested nicotine at different concentrations and up to 10 µg/mL. Our data demonstrated that the optimum activity was reached at 4 µg/mL in macrophages. The optimum concentration of nicotine was determined based on maximum activity in macrophages with least cytotoxic effect. We also studied the effect of nicotine at different time intervals ranged from 10 min to 48 h. We determined that 24 h of nicotine treatment to provide optimum response in macrophages especially when included in experiments that involve additional treatment steps such as infection or antagonists.

Recent reports suggested the involvement of TLR2 in MTB infection especially with cell wall derivatives [33,34]. Other study showed that TLR2-deficient mice failed to develop immune response against MTB [35]. However, *TLR4*-deficient mice were active and responded properly during MTB infection [36]. In Gram-negative bacterial infection and due to LPS molecules, *TLR4* was reported to play a role in recognition of enteric pathogens [34]. In this study, we investigated the interaction of MAP, a CD-associated pathogen, with both TLR2 and *TLR4*. We observed a significant increase in TLR2 expression and, in consequent cytokines including *TNF-α* and *IL-8* in MAP-infected macrophages, there was no change in *TLR4* expression in the same cells (Figure 1). In macrophages treated with TLR-2 antagonist, we observed a significant decrease in expression of *IL-8* and *TNF-α* (Figure 4). We concluded that MAP relies on TLR2 during infection at least in our macrophage system. In this study we also blocked *TLR4* with *TLR4*-IN-C34 antagonist which did not affect the inflammatory response in MAP-infected macrophages, which confirms that MAP interaction with TLR2 is key during infection. Similarly, there was limited immune response in LPS-treated macrophages blocked with TLR-4 antagonist which confirms that Gram-negative bacteria rely primarily on *TLR4* during infection (Figure 3 and Figure 4). This finding is consistent with observation by other group who reported that TLR-4 knockout mice are resistant to Gram-negative bacterial infection [37].

In our efforts to understand the effect of cigarette smoking on CD patients, we investigated possible interaction between TLR2 and nicotine in CD-like macrophages. The latter included activated macrophages infected with MAP or MTB or treated with LPS to represent Gram-negative bacteria. Initially, we did not observe any change in the expression of either TLR2 or *TLR4* in uninflected cells under nicotine exposure. However, in MAP-infected macrophages, nicotine has significantly decreased expression of both TLR2 and *TLR4*. Although similar observations were reported in MTB-infected macrophages derived monocyte (MDMs) and in lung epithelial cells [34,38], this is the first data to show a negative effect for nicotine on TLR2 expression in macrophages infected with MAP. We are still uncertain about the impact of this observation on MAP viability and burden in macrophages. We speculate that it may reduce cellular apoptosis, increase retention of bacterial load and ultimately increase inflammatory response. The finding should provide significant insights toward understanding the effect of cigarette smoke on worsening inflammation symptoms in CD patients.

To understand how nicotine modulates TLR-2 in CD macrophages, we investigated TLR2 downstream signaling in infected macrophages under nicotine stress. Specifically, we characterized the effect of nicotine on MyD88 in association with TLR2 in presence and absence of infection. MyD88 was reported to play an essential role in the development of early innate immunity against acute MTB infection [39]. This was based on a defective immune response against MTB in MyD88-deficient mice [39]. In the present study, MAP upregulated MyD88 expression and increased its specific correspondence of *IL-8* (Figure 2). Surprisingly, nicotine has further increased MyD88 and *IL-8* expression in MAP-infected macrophages. This suggest that other TLRs other than TLR2 or *TLR4* may be involved in this response. We speculate that TLR9 may be involved in this response based on an earlier report of an MTB infection model [40]. Whether TLR9 is involved alone or in interaction with other factors remains unclear. We believe that TLR9 might interact with TLR2 because of a report by other investigators who observed a shared phenotypic characteristics of MTB infection in TLR2 and TLR9 double-deficient mice and of MyD88-deficent mice [41]. This study illustrates the crucial role of MyD88 in upregulation of pro-inflammatory genes during MAP infection. Macrophages treated with MyD88 antagonist resulted in decreasing in *IL-8* and *TNF-α* levels to level similar to uninfected cells (Figure 5). We also observed a synergistic effect between MyD88 antagonist and nicotine as illustrated by shifting macrophages polarization to M2 and increasing *IL-10* during MAP infection. In the same manner nicotine decreased the *iNOS* and *IL-8* levels to minimum after TLR2 blockade in the same cells (Figure 6). Collectively, these findings led us to propose that MAP-Nicotine interaction with TLR-2 during MAP infection is MyD88-dependent, the opposite is true for TLR-4. Moreover, when TLR2 or MyD88 were blocked with antagonists, there was no significant change in MAP load in macrophages even after 48 h exposure to nicotine treatment (Figure 7). This observation suggests a key role for TLR2 and MyD88 in MAP recognition and cytokine production but not in MAP uptake and internalization in macrophages.

While MAP and LPS represent most pathogens associated with CD pathogenesis, there is no doubt that individual microbiome content plays a major factor in CD inflammatory episodes and symptoms flares. Recent studies of the microbiome in patients with CD revealed a shift in the gut microbial populations [42,43]. Specifically, it was reported that CD patients’ microbiome shows an increase in *Enterococcus* spp. and *Bacteroides* spp. and a decrease in Firmicutes *(Clostridium coccoidos*, *Eubacterium rectale*, and *Clostridium leptum*) and Actinobacteria (high guanine and cytosine content bacteria (Propionibacteriaceae and Bifidobacteria)) compared to healthy controls [44]. One study reported a specific variation in TLR2 and *TLR4* expression on lamina propria cells in CD patients compared to healthy controls [42,43]. The effect of cigarette smoke and nicotine on intestinal populations other than MAP- and LPS-containing microorganisms have not been investigated. It is important to acknowledge that the effect of nicotine on microbial community might be different as we reported in this study on individual infections. More studies are needed to understand how cigarette smoke may affect microbial load, content and interaction with macrophages in CD smokers.

In summary, we showed that, in MAP-infected macrophages, nicotine has significantly decreased expression of both TLR2 and *TLR4*. MAP upregulated MyD88 expression and increased its specific correspondence of *IL-8*. Surprisingly, nicotine has further increased MyD88 and *IL-8* expression in MAP-infected macrophages. Thus, this study illustrates the crucial role of MyD88 in upregulation of proinflammatory genes during MAP infection. Collectively, we suggested that MAP-Nicotine interaction with TLR-2 during MAP infection is MyD88-dependent. Moreover, when TLR2 or MyD88 were blocked with antagonists, there was no significant change in MAP load in macrophages even after 48 h exposure to nicotine treatment.

Overall, the data is intriguing about the role of TLR2 and MyD88 signaling in MAP-infected macrophages in association with nicotine exposure. More studies are needed to expand on our findings. Specifically, it is important to validate our findings in primary macrophages from CD patients with and without MAP infection. Ultimately, it would be the most informative to validate our findings in mice during infection and under cigarette smoking conditions.

## Figures and Tables

**Table 1:** Toll-like receptors (TLRs) and their signaling pathways.

TLR	Functional Architecture	Location and Ligands
TLR1	Heterodimer with TLR2	Extracellular binds with triacylated lipopeptide
TLR2	Heterodimer withTLR1/6	Extracellular bind with mycobacterial components
TLR3	Homodimer	Intracellular binds with nucleic acids
TLR4	Homodimer	Extracellular binds with LPS or HSP
TLR5	Homodimer	Extracellular binds with bacterial flagellin
TLR6	Heterodimer with TLR2	Extracellular with diacylated lipopeptides
TLR7	Monomer	Intracellular binds with viral nucleic acids
TLR8	Monomer	Intracellular binds with viral nucleic acids
TLR9	Homodimer	Intracellular binds with bacterial CpG DNA

TLR: toll-like Receptor, LPS: lipopolysaccharides.

**Table 2:** Receptor antagonists’ names, functions and their molecular structures.

Antagonist Name	Function	Molecular Structure
TLR4-IN-C34	An aminomonosaccharide that **inhibits TLR4 signaling** by docking with the hydrophobic pocket of the TLR4 co-receptor	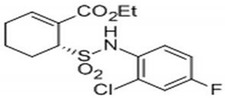
MMG11	***TLR2*****antagonist**: exhibits selectivity for TLR2 over TLR4,5,7,8, and 9	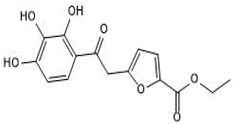
T6167923	**Novel inhibitor of*****MYD88***: disrupts MyD88 homodimeric formation, which is critical for signaling function	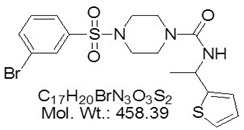

**Table 3:** RT-PCR primer sequences for interested genes.

Gene	Forward Primer Sequence (5′ → 3′)	Reverse Primer Sequence (5′ → 3′)
***GAPDH***	5′-CTTTTGCAGACCACAGTCCATG-3′	5′-TTTTCTAGACGGCAGGTCAGG-3′
**TLR2**	5′-ACGATATGCTGTCAAACACAATGACTTA-3′	5′-TGTTGCTAATGTAGGTGATCCTGTTG-3′
***TLR4***	5′-AGAGAAGACACCAGTGCCTCA-3′	5′-AGTGAGAAGTTCTGGGCAGAAG-3’
**MyD88**	5′-GGAGATGGACTTTGAGTACTTGGAGAT-3′	5′-CCCAGCTTGGTAAGCAGCTC-3′
***iNOS***	5′-GGAGCAACGTTGAGGAAATAAGACT-3′	5′-AAGAGCCAGAAGCGCTATCAC-3′
***IL-8***	5′-CACCGGAGCACTCCATAAGG-3′	5′-AAAAAGGATGTTTGTTACCAAAGCATCAA-3′
***TNF-α***	5′-TCTCCCTCAAGGACTCAGCTTTCTG-3′	5′-TGAGAGGAAGAGAACCTGCCTGG-3′
***MMR***	5′-GGAGGATTCCATGTATTTGTGAGC-3′	5′-AAATGAGTGAAGTGAAATCAGTTACCT-3′
***IL-10***	5′-ATGTCTAGTTCAGGCAGTCCCA-3′	5′-GGGCTTGCTCTTGCAAAACC-3′
